# Case Report of Prescribing in Attention Deficit Hyperactivity Disorder With Glycogen Storage Disease Type 1A

**DOI:** 10.1192/bjo.2024.659

**Published:** 2024-08-01

**Authors:** Mahmoud ElGhandour, Roop Gill, Louise Morganstein

**Affiliations:** Central North West London NHS Trust, London, United Kingdom

## Abstract

**Aims:**

Attention Deficit Hyperactivity Disorder (ADHD), is a neurodevelopmental condition affecting both children and adults, with a global prevalence estimated to be around 5% in children and 2.5% in adults, significantly impacting daily functioning, academic performance, and interpersonal relationships.

Glycogen Storage Disease Type 1A (GSD1A) is a rare metabolic disorder that occurs in approximately 1 in 100,000 births. It is characterized by accumulation of excessive glycogen and fat in the liver and kidneys that can result in growth retardation.

The aim is to increase knowledge of pharmacological management of ADHD in patients with GSD1A.

**Methods:**

Our patient is a 16-year-old boy with both diagnoses of GSD1A and ADHD.

GSD1A is treated with a special diet of frequent small servings of carbohydrates which must be maintained day and night throughout life, given via PEG tube.

ADHD symptoms cause functional impairment and affecting his school attainment requiring treatment. However, stimulant medication, such as methylphenidate, which are first- and second-line treatments, can cause appetite suppression that would increase the risk of fatal hypoglycaemia in GSD1A.

The literature review of case reviews with similar presentations, aiming to confirm the absence of contraindications for prescribing methylphenidate in patients with GSD1A, showed no identified contraindications, and relevant papers were not found.

Collaboration with the metabolic disorders team at Great Ormond Street Hospital was established to verify the absence of contraindications and facilitate potential adjustments to feeds if necessary.

Short-acting methylphenidate was administered to mitigate appetite suppression and enable prompt reversal of potential side effects, owing to its brief half-life. This approach also aimed to facilitate regular dietary intake.

Gradual bi-weekly dosage increments of 5mg, coupled with vigilant side-effect monitoring, lead to enhanced attention and concentration, ultimately contributing to improved school attainment.

**Results:**

Trial of short acting methylphenidate to ensure limited appetite suppression and allow opportunities for regular dietary intake. Slow dose titrations and weekly monitoring for response and side effects is vital. This young man's ADHD was successfully and safely treated.

**Conclusion:**

This case shows that with careful liaison and planning, methylphenidate can be safely prescribed to patients with GSD1A. Our experiences show that using short-acting preparations of methylphenidate initially allows slow and careful titrations.

